# Additive Manufacturing for Soft Robotics: Design and Fabrication of Airtight, Monolithic Bending PneuNets with Embedded Air Connectors

**DOI:** 10.3390/mi11050485

**Published:** 2020-05-09

**Authors:** Gianni Stano, Luca Arleo, Gianluca Percoco

**Affiliations:** Department of Mechanics, Mathematics and Management, Polytechnic University of Bari, Via E. Orabona 4, 70125 Bari, Italy; luca.arleo7@gmail.com (L.A.); gianluca.percoco@poliba.it (G.P.)

**Keywords:** additive manufacturing, 3D-printed soft robots, soft actuators, embedded air connector, 3D-printed air tight actuator, pneumatic actuator

## Abstract

Air tightness is a challenging task for 3D-printed components, especially for fused filament fabrication (FFF), due to inherent issues, related to the layer-by-layer fabrication method. On the other hand, the capability of 3D print airtight cavities with complex shapes is very attractive for several emerging research fields, such as soft robotics. The present paper proposes a repeatable methodology to 3D print airtight soft actuators with embedded air connectors. The FFF process has been optimized to manufacture monolithic bending PneuNets (MBPs), an emerging class of soft robots. FFF has several advantages in soft robot fabrication: (i) it is a fully automated process which does not require manual tasks as for molding, (ii) it is one of the most ubiquitous and inexpensive (FFF 3D printers costs < $200) 3D-printing technologies, and (iii) more materials can be used in the same printing cycle which allows embedding of several elements in the soft robot body. Using commercial soft filaments and a dual-extruder 3D printer, at first, a novel air connector which can be easily embedded in each soft robot, made via FFF technology with a single printing cycle, has been fabricated and tested. This new embedded air connector (EAC) prevents air leaks at the interface between pneumatic pipe and soft robot and replaces the commercial air connections, often origin of leakages in soft robots. A subsequent experimental study using four different shapes of MBPs, each equipped with EAC, showed the way in which different design configurations can affect bending performance. By focusing on the best performing shape, among the tested ones, the authors studied the relationship between bending performance and air tightness, proving how the Design for Additive Manufacturing approach is essential for advanced applications involving FFF. In particular, the relationship between chamber wall thickness and printing parameters has been analyzed, the thickness of the walls has been studied from 1.6 to 1 mm while maintaining air tightness and improving the bending angle by 76.7% under a pressure of 4 bar. It emerged that the main printing parameter affecting chamber wall air tightness is the line width that, in conjunction with the wall thickness, can ensure air tightness of the soft actuator body.

## 1. Introduction

Soft robotics is an emerging scientific field conceptually conflicting with traditional hard robotics, the latter is typically characterized by (i) rigid interconnected links that can move in predetermined environments, (ii) robots that are typically heavy and expensive, and (iii) a high level of control due to expansive electronics. In contrast, soft robotics (i) typically employs continuum bodies which can function in a wide range of unknown environments, (ii) have low weight and cost, and (iii) have a low level of control [1].

Soft materials are key to manufacturing soft robots. This material class provides several benefits such as the possibility to passively adapt the robot body to the surrounding environment, a high impact resistance, and the possibility to design robots with very complex geometries and shapes [2]. Biological organisms (e.g., muscle tissue, fat, or skin) are composed of materials with Young moduli ranging from 10^4^ to 10^9^ MPa, hence all materials with a Young modulus in that range could be defined as soft materials [3]. An important feature of soft materials is their viscoelasticity; in fact, many soft materials found both in nature and in soft robotics are viscoelastic. These soft materials (e.g., PDMS, hydrogel, and urethane) dissipate energy while maintaining a stable motion when a load is applied [4].

Animals and biological organisms can perform complex movements with soft structures and can adapt to several environments, which is why they often serve as a source of inspiration for researchers who try to apply biological concepts to the field of soft robotics [5]. A milestone in the field of bioinspired soft robotics was the development of a soft robot without rigid structures, inspired by *octopus vulgaris* and following the octopus’ muscular hydrostat principle [6]. The octopus’s morphology was replicated using cables and shape memory alloys (SMA) springs for actuation, thereby obtaining the classic complex movements of the octopus such as bending along four directions, elongation, and shortening. Another interesting bioinspired work [7] consists of a soft robot named GoQBot which mimics the caterpillar rolling behavior through ventral flexion among two adjacent geometric features. This was achieved by implementing silicon rubbers as soft materials for the caterpillar body and SMA coils as actuators. Two of the most attractive applications for soft robotics are in the fields of manipulation and biomedicine [8,9,10]; in particular, soft robots used for surgery can be assisted by innovative electromagnetic tracking systems for surgical navigation [11,12]. In accordance with [13], it is possible to divide soft robots based on the functionality of their actuation system into three categories: (i) variable length tendon (split into two subclasses: tension cable and SMA); (ii) fluidic actuation (split in two subclasses: pneumatic and hydraulic), and (iii) electroactive polymers (EPA) (split in two subclasses: electronic and ionic). Among the soft robots based on fluidic actuation, an emerging class is called PneuNet (pneumatic networks). Their working principle is very simple: they are made of soft materials and contain several pneumatic chambers that are interconnected via channels with actuation typically being achieved through compressed air, forcing the chambers to expand and change their shape. PneuNets movements directly depend on their design, e.g., an extensible top layer (equipped with pneumatic chambers) overlaying a rigid bottom layer leads to a bending motion. Without the inextensible bottom layer one obtains a lengthening or extending motion [14,15,16]. At the state of the art, despite a growing interest in the last few years in 4D printing soft robots [17,18] and recent advances in 3D-printing composite-based soft actuator [19], additive manufacturing (AM) aimed to the production of soft actuators is still underexploited: low cost AM technologies, such as fused filament fabrication (FFF) are mainly used for rapid tooling [20,21,22,23,24], while only more expensive silicone extrusion [25,26], polyJet technology [27], and stereolithography [28] are used for direct rapid manufacturing of the whole actuator.

In this paper, the feasibility of manufacturing a monolithic bending PneuNet (MBP) actuator with FFF technology by replacing the classic manufacturing process based on the elastomeric rubber molding with the melting extrusion of flexible filaments made by thermoplastic polyurethane has been investigated. The advantages offered by FFF technologies in soft robotics are: (i) reduction of assembly tasks and (ii) increase of automation degree during the manufacturing process. This is achieved by designing a 3D-printed embedded air connector (EAC) to be embedded into the soft robots produced by FFF. This novel and innovative EAC is completely air tight and embeds the male air connector, assuring compatibility with standard female air connectors. Indeed, the EAC is manufactured in the same printing cycle of the soft robot and does not require any assembly. Several design and process parameters have been tested to find the optimal printing orientation that ensures complete air tightness.

Four different monolithic bending PneuNet (MBP) shapes have been experimentally studied to find the one exhibiting the best bending angles and flexibility and identify the design parameters that affect bending performance. Finally, a way to improve bending performance (the bending angle was improved by 78.7%), working on the best MBP shape, has been found by examining the relationship between printing parameters and constitutive design elements and proving how the Design for Additive Manufacturing (DfAM) approach is useful in applications like these. In summary, the workflow of this research can be outlined as follows: (i) manufacturing of a leakage-free air connector, (ii) identifying the most performing actuator shape with embedded air connector, and (iii) improving the performances of the best actuator shape in terms of flexibility.

## 2. Materials and Methods

### 2.1. Materials

The dual-extruder 3D printer Ultimaker 3 (Ultimaker, Utrecht, the Netherlands), which enables the usage of two different filaments in the same printing cycle, has been used to manufacture the monolithic bending PneuNet (MBP). The two commercial filaments chosen were: (i) polyurethane thermoplastic produced by Ultimaker with a shore A hardness of 95 (TPU 95A), a tensile modulus of 26 MPa, and an elongation at break of 580% and (ii) a low-friction polyurethane thermoplastic developed by BASF (Ludwigshafen, Germany) and on the market since 2019 with a shore A hardness of 80 (TPU 80A LF) and a tensile strength ranging from 17 to 22 MPa depending on the printing direction of the sample. All technical data were from filament’s datasheets. TPU 95 A was used to create the male embedded air connector and to fabricate the inextensible portion of the actuator, whereas TPU 80 A LF was chosen to manufacture the extensible segment of the MBP.

The ideal material to manufacture the air connector was rigid and does neither break nor twist under high pressure (up to 7 bar). At the same time, boundary interface problems between the two materials with very different hardness values must be taken into account during the dual-extruder printing process.

As demonstrated by Lopes et al. [29], the tensile strength in specimens made of rigid and soft materials, namely, TPU and polylactic acid (PLA), is lower than that of specimens made of TPU only or PLA only. This behavior demonstrates the weak affinity between TPU and PLA. Additionally, breakage of the specimens made of TPU and PLA always occurred at the interface between both materials. Based on these results, it was decided to use two TPU-based filaments: for the rigid (TPU95A) and soft/deformable (TPU 80 LF) parts of the MBP.

### 2.2. Leakage-Free 3D-Printed Embedded Air Connector—First Printing Orientation

A common problem with pneumatic soft-robots typically occurs at the interface between the soft robot and the pneumatic system that supplies the compressed air, where recurrent air leaks may not only reduce the soft robot performance but may also pose a potential danger for the surrounding environment once high pressures are reached. This problem is often addressed through solutions that are rather complex from an assembling point of view [23,30].

**Figure 1:**
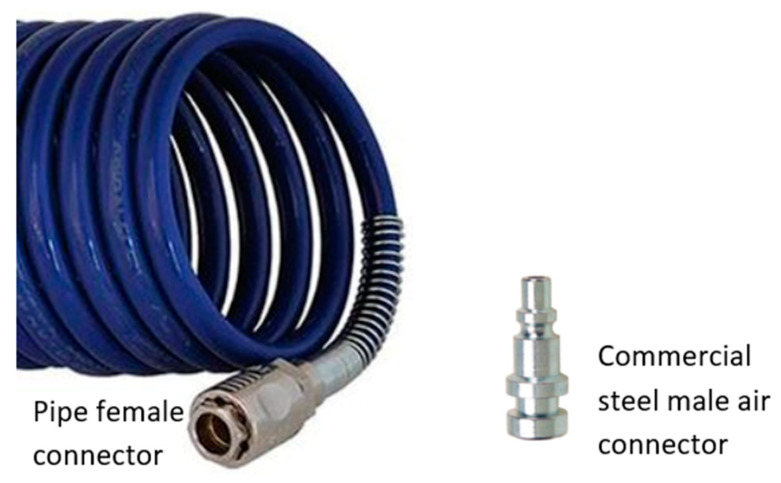
Pneumatic pipe and commercial air connector.

**Figure 2:**
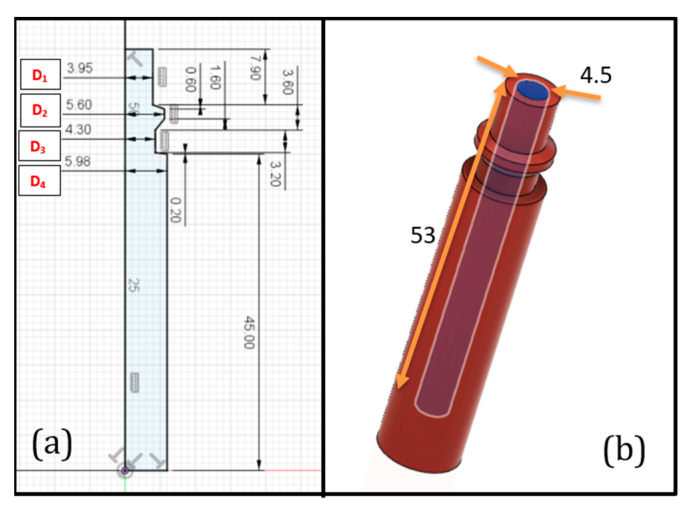
Revolved sketch (**a**) (all dimensions are in mm) and (**b**) 3D model of the embedded air connector (EAC).

Here, an innovative 3D-printed male embedded air connector (EAC) which can be easily embed in every soft robot made with FFF technology requiring only one printing cycle has been developed. The proposed EAC ensures the total absence of any air leaks at the interface between the soft robot and the pneumatic system. Its general purpose is to mimic the commercial male air connectors (often made of steel), quickly engraftable into the female connector attached to pneumatic pipe (Figure 1). Since 3D-printed soft robots are made by soft materials, it has been decided to use as well a soft material for the EAC (it will be manufactured in the same printing cycle of the soft robot) in order to reduce several manufacturing problems, as explained above. The EAC was initially fabricated and tested with the same nominal size as the commercial male connector but the tests highlighted air leaks. Due to the lack of scientific literature addressing this issue, a trial and error method was applied to find the correct nominal value of the connector’s diameter, increasing its value. The four external diameters of the EAC (from D_1_ to D_4_ in Figure 2) were increased in steps of 0.1 mm until the air tightness was reached. The EAC diameters needed to be designed at least 0.5 mm wider than those of the standard connectors.

**Figure 3:**
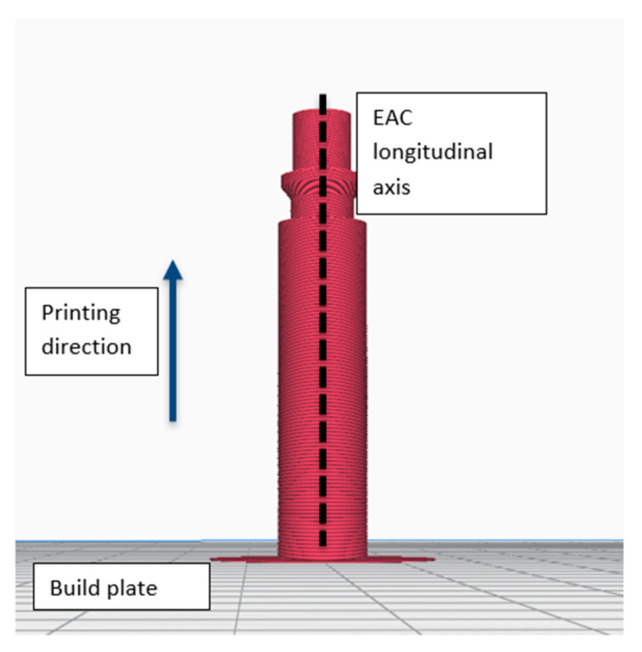
Printing orientation.

Ultimaker Cura 4.4 software (Ultimaker, Utrecht, the Netherlands) was used to set the printing parameters and communicate with the 3D printer. Table 1 shows the manufacturing parameters used to fabricate the EAC. Because of its circular shape, the EAC was printed with its longitudinal axis perpendicular to the build plate (Figure 3) which afforded the following advantages: (i) no supports are required and (ii) the nozzle can perform circular movements to create the EAC shape.

This printing orientation also guarantees the complete absence of air leaks.

The printed EAC was inserted into the pneumatic pipe and several tests were performed to evaluate its air tightness. For each test, the EAC was submerged in a beaker filled with water to facilitate the detection of air leaks. To work safely, EAC was anchored to the beaker trough a custom-made support. Each EAC underwent the following 3 tests: (i) the pressure was increased in steps of 1 bar from 0 to 7 bar (uphill phase) and then reduced again in the same manner to reach 0 bar (downhill phase), with 1 min between pressure changes. This test was repeated 10 times for each specimen; (ii) here, the pressure abruptly alternated between 0 and 7 bar, remaining for 5 s at each level and repeating these cycles 50 times; and (iii) here, the specimens were exposed to a pressure of 7 bar for a time of 15 min.

During each of the above tests, no water bubble was detected into the beaker full of water, thus demonstrating the absence of any air leaks.

The innovativeness of the EAC consists in a single-step printing cycle to embed connectors potentially in every soft robot made by FFF, reducing the use of commercial solutions, often unsuitable for pneumatic soft robots.

### 2.3. Leakage-Free 3D-Printed Embedded Air Connector—Second Printing Orientation

**Figure 4:**
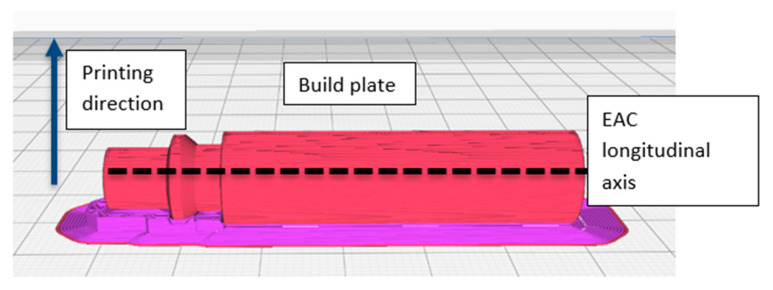
Second printing orientation; the purple parts are the supports made from polyvinyl alcohol (PVA).

In this section, one alternative printing orientation has been examined (Figure 4): with this orientation (EAC longitudinal axis parallel to the build plate), supports were required during the printing process and the staircase effect affected heavily the cylindricity of the component showing a high dependence on the layer height. These two features affect the EAC’s air tightness. In particular, the supports can damage the EAC when they are manually removed from the structure and thus cause air leaks. For this reason, the effect of two different kinds of materials for the supports has been studied: TPU 95 A (the same used for the EAC), which requires manual removal and polyvinyl alcohol (PVA) (Ultimaker, Utrecht, the Netherlands), a water-soluble material that does not require any manual removal.

Regarding the cylindricity, it has been decided to investigate the staircase effect on EAC air tightness. Layer heights considered in this study were 0.15 mm (the same used in the first printing orientation) and 0.05 mm, the minimum layer height allowed by the Ultimaker 3 3D printer.

Using the support material and layer height as factors and assigning two levels to each factor, a full 2^2^ factorial experiment has been performed to understand the influence of these factors on air tightness and if a combination of factors that ensures the absence of air leaks exists. The response variable was the leakage (L/min) measured with the following method:

1. The EAC is connected to the pneumatic pipe of the compressor and a pressure $P_{2}$ of 3 bar has been reached.
2. The air supply is stopped and the amount of pressure reduction (indicative of an air leak) is measured, resulting in the time $t_{drop}$ until a new pressure $P_{1}$ of 2 bar is reached.
3. Compressed air is resupplied and the time to reach $P_{2}$, $t_{rise}$, is measured.

The amount of air leakage ($q_{leak}$) in L/min can then be calculated as

(1) $$q_{leak}=\;Q_{c}*\frac{t_{rise}}{t_{rise}+t_{drop}}$$

where $Q_{c}$ is the air flow of the compressor (180 L/min).

The software Minitab 17 was used to analyze the 2^2^ factorial experiment. Table 2 summarizes the nomenclature of the factors and levels.

**Figure 5:**
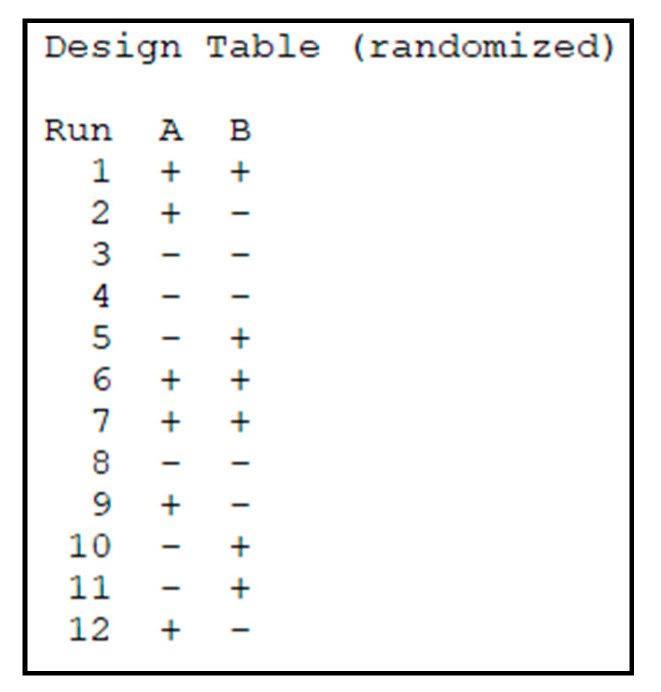
Minitab 17 screenshot where the randomized manufacturing plan is shown.

Each EAC is characterized by a certain combination of factors and levels and was printed in triplicate (number of replication (n) of the factorial plan is 3) to account for variabilities in the manufacturing process and obtain a better estimate of the impact of factors. To reduce the effect of uncontrollable external factors related to the 3D printing process, the manufacturing of the EAC samples was completely randomized by using the fabrication plan (Figure 5).

Table 3 shows the amount of air leakage ($q_{leak}$) for each sample as well as the overall mean µ and variance $\sigma^{2}$ for each factor combination (Table 3).

The mean, µ, and variance, $\sigma^{2}$, are calculated as follows:

(2) $${µ}_{j}=\frac{1}{n}\overset{n}{\underset{i=1}{\sum }}{\left(q_{leak}\right)}_{i,j}$$

(3) $$\sigma^{2}=\frac{1}{n}\overset{n}{\underset{i=1}{\sum }}{\left[{\left(q_{leak}\right)}_{ij}-{µ}_{j}\right]}^{2}$$

where n is the number of replicates, j is the factor combination with j=(1), a, b, ab, and ${\left(q_{leak}\right)}_{i,j}$ indicates the amount of air leakage for the i−th replicate and j−th factor combination.

**Figure 6:**
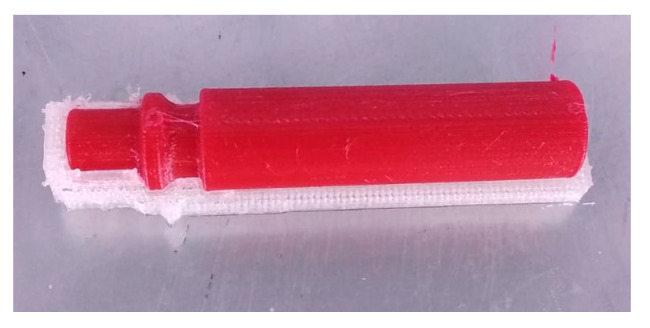
EAC with PVA support.

For the combinations ab and b the variance is 11.3 and 0.29, respectively, the highest variance among the 4 combinations. Both combinations ab and b are characterized by the same level of factor B, namely, the usage of TPU 95 A as support material. Manually removing the TPU 95 A supports is a critical operation that requires a high level of skill of the operator to avoid damage to the EAC which may explain the high level of variance associated to the samples using TPU 95 A support materials. In fact, for these two combinations, the amount of air leakage was closely related to the support removal. Combinations (1) and a used PVA support materials and only showed low variances of 0.001 and 0.02, respectively, because no manual operations were requested to remove the supports as they are dissolved in water. Figure 6 shows an EAC specimen with PVA support.

**Figure 7:**
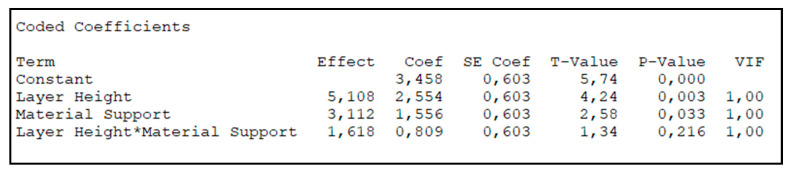
Results of the factorial plan.

Several conclusions can be drawn from the data analysis (Figure 7):

1. No combination resulted in complete air tightness when the EAC was printed with its longitudinal axis parallel to build plate.
2. The main parameter affecting air leakage is layer height which indicates that switching from a layer height of 0.05 to 0.15 mm resulted in a greater increase in air leakage than changing the support material.
3. The type of support material only has a minor effect on air leakage. Keeping the layer height constant, the variance increases by moving from PVA to TPU 95 A which is due the latter material requiring the manual removal of the support.
4. The interaction between both parameters is small in comparison to the effect of individual parameters. Additionally, in accordance with *p*-values, it is possible to assert that the main effects of A and B are statistically significant and that there is no interaction among them.
5. The best solution in terms of minimizing air leakage is also the most expensive one because it requires the use of two different materials and the amount of extruded filament is larger than with other combinations (the quantity of extruded filament increases when the layer height decreases). The cost, as estimated by the slicing software, for the four combinations a, b, ab, and (1) is 0.74, 0.61, 0.58, and 0.89 €, respectively.

In conclusion, if the EAC is printed with its longitudinal axis perpendicular to the build plate, it results in complete air tightness. If the EAC is printed with its longitudinal axis parallel to build plate, some air leakage occurs, which can be minimized to a mean value of 0.157 L/min, however, by using tailored strategies (PVA as support material and a layer height of 0.05 mm).

## 3. Results and Discussion

In this section, four different monolithic bending PneuNet (MBP) shapes to find the best shape in terms of flexibility have been analyzed. First of all, a manner to fabricate MBPs to ensure air tightness has been presented. Afterwards, each MBP has been characterized by relating the bending angle and the tip position to the input pressure and certain rules that link the design constitutive parameters to the bending behavior have been derived. Finally, different design and manufacturing parameters for the MBP with the best bending performance (R-type) have been varied to further improve its flexibility.

### 3.1. Monolithic Bending PneuNet (MBP)—Shape Investigation

As explained in Section 1, PneuNet (pneumatic network) is a class of soft actuator which can perform several movements in accordance with its design geometry. In this paper, the possibility of 3D printing of a monolithic bending PneuNet (MBP) with the EAC directly embed into the structure (i.e., without using any commercially available air connectors) has been investigated. The classic bending PneuNets are composed of two different portions, typically referred as “extensible layer” and “inextensible layer.” However, the authors prefer to refer to them as “extensible portion” and “inextensible portion” to avoid possible confusion with the term “layer” used in the context of FFF technology where it refers to the extruded filament deposited by the nozzle. The core of the extensible portion consists of a system of pneumatic chambers interconnected via several channels. When the chambers are pressurized, they expand resulting in a change of their shape. Because the extensible portion is deformable, it is always made by soft materials. The inextensible portion is more rigid but still allows bending which is achieved from the differential strain effect due to the different softness of the two portions.

Because elements made using FFF technology are anisotropic (it is difficult to predict their behavior using classical methods such as Finite Element Analysis simulations), and also fabricating MBPs with this technology is inexpensive (the total cost for each MBP is less than 5 €) and automated (no manual tasks are required as for PneuNets manufactured with molding technologies), it has been decided to manufacture and empirically evaluate 4 differently shaped MBPs with the aim to find the shape that shows the best performance in terms of bending angle.

**Figure 8:**
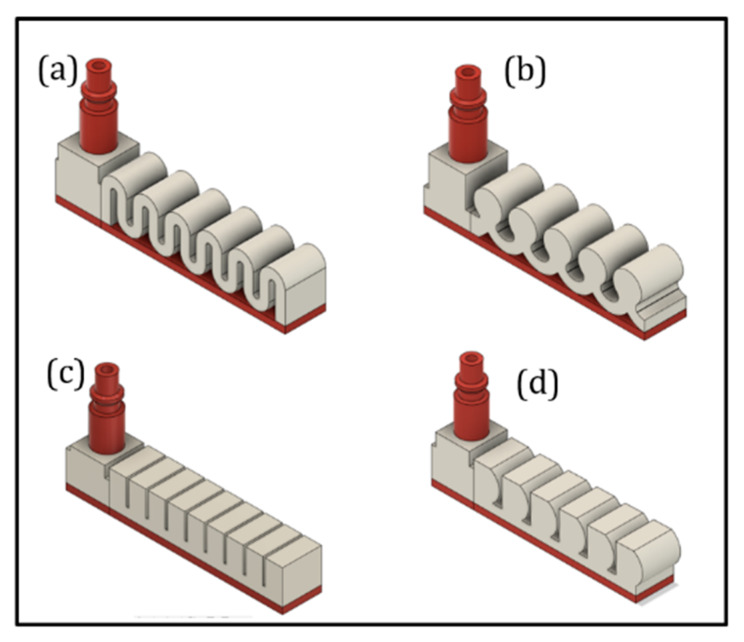
MBP shapes: (**a**) S-type, (**b**) B-type, (**c**) R-type, and (**d**) D-type.

Each MBP consists of an EAC, an embedded L-junction to direct air flow to the extensible portion, an inextensible portion with a height of 3 mm, and an extensible portion equipped with several pneumatic chambers. The difference between the 4 MBPs is limited to the shape and geometry of the extensible portion (Figure 8). While each MBP has the same width of 18 mm, the active bending length varies slightly to allow a finite pattern number of chambers. The active bending length (or in other terms the length of the extensible portion) for R, D, B, and S-types is 80, 76.9, 76, and 81.4 mm, respectively.

Design for additive manufacturing (DfAM) is a technique based on the idea to design and optimize a product which will be manufactured through AM techniques in order to improve its properties [31]. An easy-to-use DfAM conceptual framework that is divided into five phases was presented in [32].

**Figure 9:**
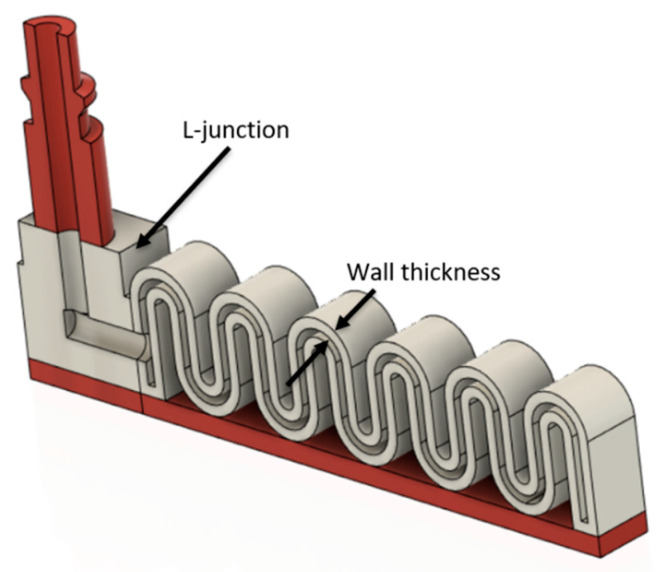
Cross section of S-type.

**Figure 10:**
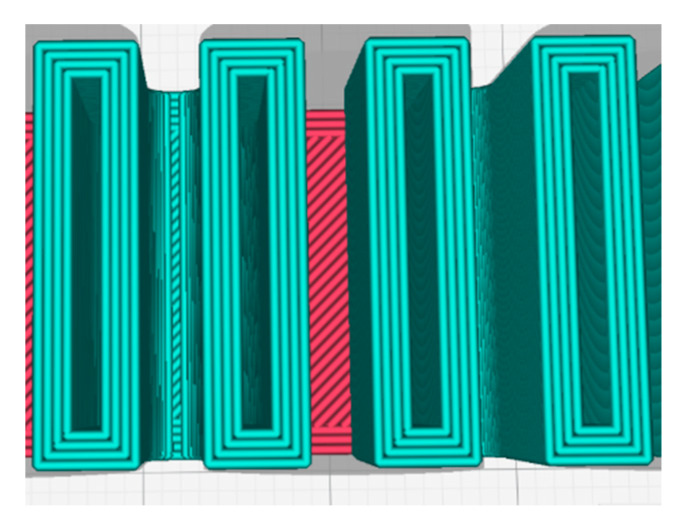
Top view of the S-type into the slicing software, it is possible to see the four adjacent lines of extruded materials; each one has width of 0.4 mm, which compose the 1.6 mm thickness of the chamber walls.

The proposed MBPs have been designed using the DfAM approach:

1. The EAC embedded in the soft actuator structure has been designed for printing with its longitudinal axis perpendicular to the build plate to ensure air tightness at the pneumatic pipe interface. With this design choice, it is necessary to direct the air flow toward the extensible portion which is achieved through an embedded L-junction that can switch the air flow from the EAC to the pneumatic chambers (Figure 9).
2. Apart from ensuring the absence of air leakage at the interface between EAC and pneumatic pipe, the authors also made sure that there is no leakage into the extensible portion. This is crucial for finding a suitable thickness of the pneumatic chamber walls (Figure 8). Both portions were fabricated using a nozzle diameter of 0.4 mm; for this reason, in the slicing software Ultimaker Cura 4.4, the line width parameter was set to 0.4 mm. For this reason, the thickness of the pneumatic chamber walls will be a multiple of 0.4 mm. Through trial-and-error method, it has been found that the minimum chamber wall thickness to ensure air tightness is 1.6 mm. Hence, the minimum number of adjacent lines of extruded filament needed to avoid air leakage is 4 (Figure 10).

As described in Section 2.1, both the rigid portion and the EAC were manufactured using TPU 95 A, whereas the extensible portion and L-junction were made of TPU 80 A LF. For both materials, a nozzle diameter of 0.4 mm was used. Table 4 lists the most important process parameters for each MBP component. The printing temperature of the TPU 80 A LF was raised from the supplier’s suggested temperature of 230 to 240 °C to improve adhesion between adjacent lines and layers: as shown in [33], an increase in printing temperature lead to an increase of manufacture strength; in pneumatic applications, it means increasing actuator air-tightness and reducing the probability of damage under high pressure. In addition, to improve the adhesion between MBPs and build plate and avoid some common AM issues such as the warping problem, a very high build temperature of 60 °C was set and the glue was used. The same layer height of 0.1 mm and line width of 0.4 mm were used for the two different materials. All four MBPs were printed using the same orientation with the inextensible portion flat on the build plate and the longitudinal axis of EAC perpendicular to the build plate. The building time and cost for R, D, B, and S-type estimated by slicing software were, respectively, 7 h 44 min, 8 h 55 min, 8 h 1 min, and 9 h 35 min and 4.11, 4.68, 4.23, and 4.99 €.

**Figure 11:**
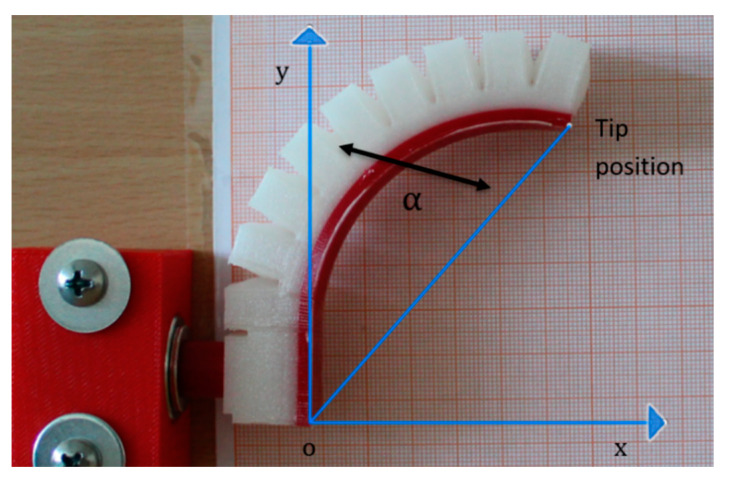
Illustrating how bending angle and tip position were calculated.

After fabrication, the four MBPs were tested to link bending angle and tip position in 2D space to pressure input. The setup consisted of an air compressor, a rigid frame to which the MBP was attached in front of a square millimeter grid used for the optical readings, and a Canon EOS D400 digital camera (Canon, Tokyo, Japan) to record the tip position and bending angle (Figure 11).

Starting from an input pressure of 0 bar (rest condition), the pressure was increased with step of 1 bar till it reaches the maximum value of 4 bar; at each pressure step, one image has been captured to measure the bending angle and the tip position.

**Figure 12:**
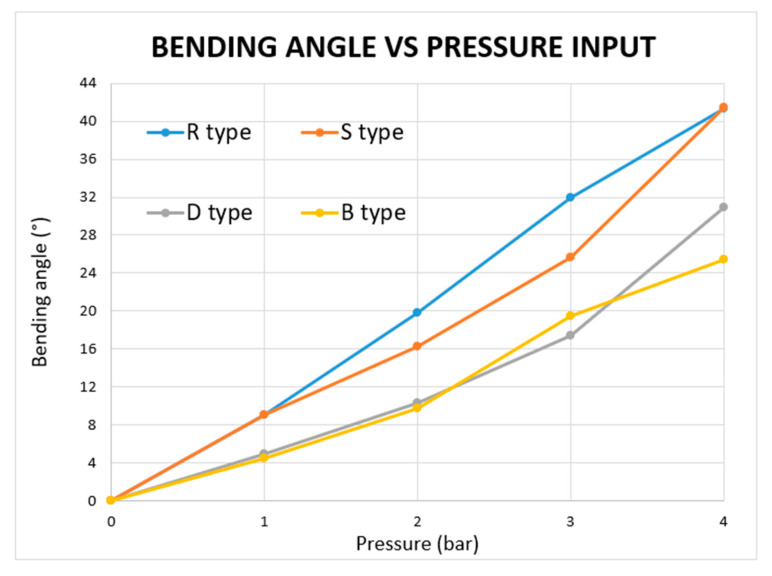
Bending angle vs. pressure for each MBP type.

Figure 12 shows the bending angles obtained for different input pressures for the four different MBP shapes. Table 5 lists the tip displacements as a function of applied pressure.

The R- and S-types were the most flexible and exhibited very similar bending angles and tip displacements. At 1 bar, their bending angles only differed by 0.1°. At higher pressures of 2 and 3 bar, the S-type is lightly more rigid than the R-type, while at 4 bar, the bending angles are again very similar (41.3° for R-type and 41.4° for S-type). D- and B-type were the least flexible and had comparable bending angles between 0 and 3 bar, while at 4 bar, the D-type was more flexible than the B-type and, however, less flexible than both R- and S-types. Overall, the R-type thus turned out to be the best MBP as it is the most flexible between 0 to 3 bar and equally flexible at 4 bar as the S-type.

From the experimental phase, several considerations about the relationship among MBPs bending behavior and constitutive design parameters can be drawn. When pressurized, the chamber walls, aligned with the air flow (in other terms parallel to air flow), tend to stretch and the walls perpendicular to the air flow (such as the top chamber wall) tend to expand.

Consequently, it is possible to translate this consideration in the maximization, for each pneumatic chamber, of the ratio (S) computed as shown in Equation (4), being $S_{par},$ the surface area of the walls parallel to air flow and $S_{per},$ the surface area of the walls perpendicular to air flow:

(4) $$\max S=\frac{S_{par}}{S_{per}}$$

**Figure 13:**
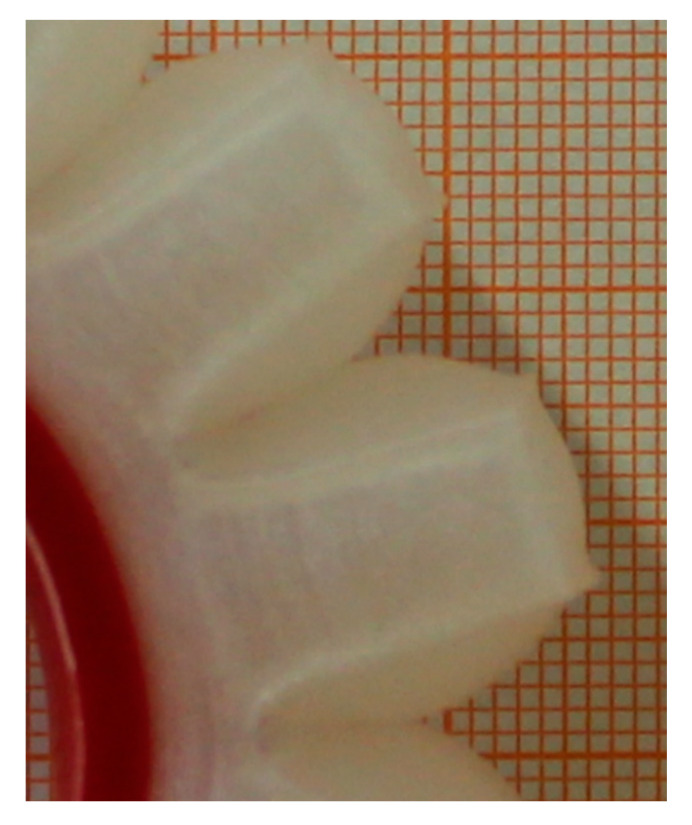
Zoom of an R-type MBP showing its behavior when pressurized.

The second most important design feature which affects the flexibility of MBPs is the close proximity of adjacent chambers. The chambers stretch their walls in the air flow direction; so, by increasing the closeness among adjacent chambers, there will be a better MBP elongation because the chambers will better push against each other. Figure 13 shows a zoom of R-type MBP during the compressed air insufflation; from this picture, it is possible to graphically see the two features described above: when compressed air is insufflate, the walls perpendicular to air flow expand themselves while the walls parallel to air flow stretch themselves and it involves the push of adjacent chambers resulting in the actuator bending movement.

For the same wall thickness and active bending length, the bending performance of the different MBPs mainly depended on their shapes; as a matter of fact, those with maximal S ratios (see Equation (4)) and proximity between adjacent chambers would perform best.

In conclusion, S- and R-types delivered the greatest bending angles because their designs optimize *S* ratio and chamber proximity. The R-type has the closest proximity between adjacent chambers, whereas the S-type has the highest S ratio.

### 3.2. Monolithic Bending PneuNet (MBP)—Improving Bending Performance

In accordance with scientific literature, the two main features affecting bending performance are the chambers’ wall thickness and the number of chambers. As regards the former, it has been proved both experimentally and theoretically that small values of wall thickness generate an increase in the final bending of the actuator [14,21]. As regards the latter, it has been shown in literature that more are the chambers for a given length, the greater is the bending [14]. In the present paper, it has been decided to focus on wall thickness.

**Figure 14:**
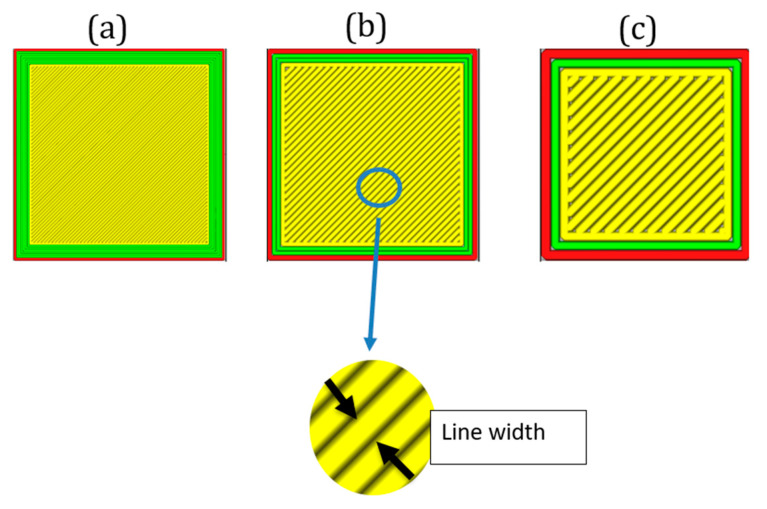
Three different line widths: (**a**) 0.2 mm, (**b**) 0.4 mm, and (**c**) 0.8 mm.

In Section 3.1, it was shown how the minimum wall thickness to guarantee air tightness was 1.6 mm, i.e., four adjacent extruded filament lines of 0.4 mm. A way to reduce wall thickness but at the same time avoid any possible air leakage has been found by working on R-type. In this study, all the design parameters, except wall thickness, have been held unchanged in order to quantify how walls thickness impacts on the bending performance. Because line width parameter (in Figure 14, it is possible to see the difference among three different line width values on the same square component) depends on nozzle size (generally this value should range from−20% up to +20% of nozzle diameter), it has been decided to use a nozzle diameter of 0.25 mm in order to set line width value lower than 0.4 mm in the slicing software (set to fabricate MBPs with wall thickness of 1.6 mm).

In this way, a line width value of 0.2 mm in the slicing software was set, and R-type wall thickness was decreased from 1.6 to 1 mm. These choices (nozzle diameter = 0.25 mm, line width = 0.2 mm, and wall thickness= 1 mm) involve five adjacent extruded lines. By testing this new kind of R-type, its air tightness has been proved.

The novel important feature discovered in this paper is that the air tightness of MBPs manufactured via FFF technology does not depend on the wall thickness as for PneuNets fabricated by molding, but it depends on the number of adjacent extruded lines that make up the chamber wall. So, the key printing parameter that needs to be tuned to avoid air leakage is “line width,” which is related to actuator design in the following way: the wall thickness of each MBP should be equal to line width value set in slicing software multiply for four or five times. This proves how DfAM is crucial for advanced applications involving FFF technology.

There is thus a direct correlation between “line width” printing parameter and MBP air tightness, and the knowledge of this relationship is the enabling key to fabricate 3D-printed soft robots with improved performance. For the 3D printer employed in this research, namely, Ultimaker 3, the smallest available nozzle diameter is 0.25 mm. For other commercial dual-extruder 3D printers, nozzles with a diameter of 0.1 mm are available; it means that it should be possible to fabricate soft actuators with values of wall thickness lesser than 1 mm (i.e., setting line width parameter as 0.1 mm and a wall thickness of 0.5 mm, air-tightness should be ensured).

**Figure 15:**
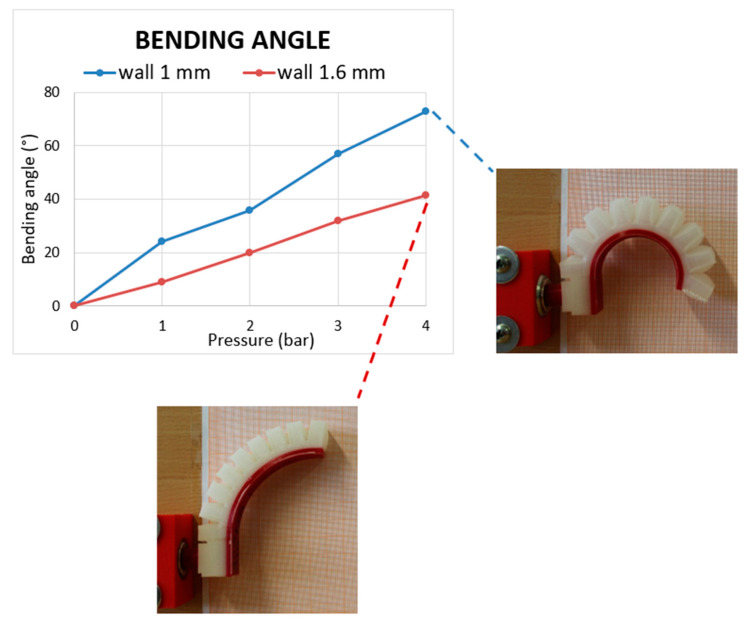
Bending angle vs. input pressure for R-type with wall thickness of 1.6 and 1 mm.

**Figure 16:**
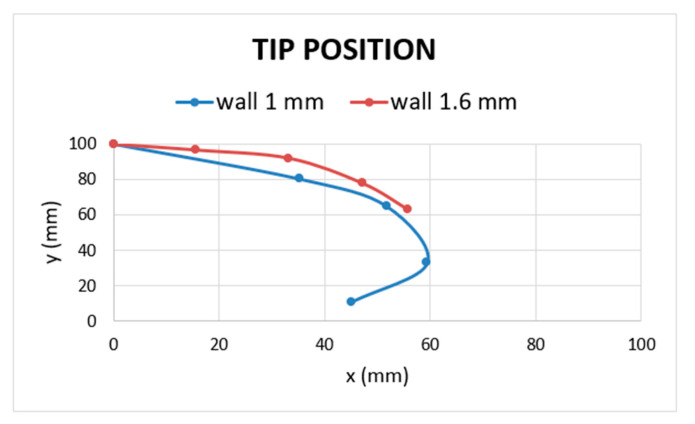
Tip position, here it is possible to see the tip movements in 2D space for R-type with 1.6 and 1 mm wall thickness.

**Figure 17:**
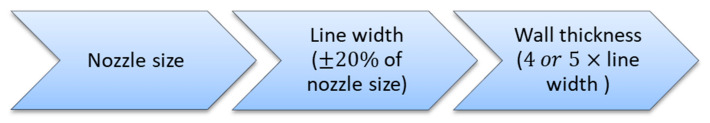
Methodology used to reduce wall thickness ensuring air tightness.

Figure 15 and Figure 16 show the effect of the wall thickness on bending angle and tip position for R-type actuator with the two different wall thickness values: 1 mm walls R-type is far more flexible than its counterpart, i.e., the 1.6 mm walls version. In fact, the improved value of bending angle when an input pressure of 4 bar was provided was 72.9° against 41.3° of the previous version, resulting in a bending angle improvement of 76.7%. Thanks to the DfAM approach, it was possible to reduce the minimum wall thickness from 1.6 to 1 mm, without air leakage, in order to greatly improve the performance of the actuator in terms of flexibility. Figure 17 illustrates the method used to reduce wall thickness, improving bending performance and ensuring air-tightness.

Starting from this new minimum thickness values, it will be possible to further increase the bending performance following two ways: (i) increasing the active bending length of the R-type from 80 to over 100 mm (generally bending PneuNets used for grasping operations have a bending length ranging from 100 to 140 mm) and (ii) increasing the number of the chambers for a given active length.

Following these approaches, optimized air-tight pneumatic actuators with further improved bending performance could be easily fabricated via FFF technology and employed in several applications (i.e., they could be used in soft grippers to manipulate objects with unconventional and unpredict shape).

This work is a first, preliminary study in the field of the soft actuators made via FFF technology, showing (i) a repeatable methodology to fabricate air-tight actuators with embedded air connector and (ii) an empirical method to choose the most performing actuator shape; future investigation are needful to reduce the gap in terms of bending performance with actuators made with traditional technologies. Although the purposed version is characterized by a bending angle of 72.9° when a pressure of 4 bar is supplied, other actuator such as the self-healing actuator of Terryn et al. [34] exhibits a similar bending angle (almost 68°) with lower input pressure (almost 2.5 bar).

## 4. Conclusions

In this paper, a new manufacturing approach for soft actuators based on the inexpensive FFF approach has been presented. A novel 3D-printed air connector with the following features was developed: (i) it can be easily embed in soft robots made with FFF technology as it can be manufactured in the same printing cycle as the soft robot, (ii) it is completely air tight at the interface between the pneumatic pipe and the soft robot, and (iii) it enables to overcome assembly problems due to the usage of marketable air connectors. Using a 2^2^ factorial plan, we could show that only one printing orientation can ensure the absence of air leaks in the 3D-printed air connector. By comparing four different 3D-printed MBP shapes, the authors found that the best performance was achieved with the R-type MBP characterized by a bending angle of 41.3° when a pressure of 4 bar was supplied. In addition, from the experimental data, the authors outlined two design rules needful to better understand why some actuator shapes result in more performance than others. In the shape selection phase, the minimum wall thickness able to ensure air tightness was 1.6 mm; by using a Design for Additive Manufacturing approach (DfAM), it has been possible to reduce the minimum wall thickness up to 1 mm, ensuring the total actuator air tightness at the same time. The new improved R-type actuator is resulted being more flexible compared to the first version: the bending angle has been improved by 76.7%, switching from 41.3 to 72.9° when a pressure of 4 bar was supplied. Line width was found to be the main parameter affecting air tightness of soft actuators made via FFF; by reducing this parameter by using nozzles with a lesser diameter (i.e., 0.2 mm), it is possible to decrease wall thickness leading to an improvement of the actuator flexibility. The authors show experimentally that the chamber walls need to consist of at least four to five adjacent extruded lines of filament to ensure air tightness. Future work might focus on improvement of MBP bending performance to reduce the gap with molded-based counterparts, which result into more flexibility (i.e., they reach a bending angle of almost 70° with an input pressure of almost 2.5 bar), by studying other parameters such as the distance between chambers, the chamber dimensions, the thickness of the inextensible portion, or the usage of more flexible materials. Furthermore, future studies could examine how to exploit the optimized MBP for several practical applications such as grasping, locomotion or rehabilitation.

## Figures and Tables

**Table 1:** Parameters for the embedded air connector (EAC).

Parameter	Value
Nozzle diameter	0.4 mm
Printing temperature	225 °C
Printing speed	25 mm/s
Infill density	100%
Infill pattern	Circular
Retraction distance	7.5 mm
Adhesion type	Brim
Layer height	0.15 mm
Line width	0.4
Bed temperature	60 °C

**Table 2:** For the factors and levels used.

	A (Layer height)	B (Support material)
**−1**	0.05 mm	PVA
**+1**	0.15 mm	TPU95A

**Table 3:** Plan 2^2^ with three replicates.

Combination Name	A	B	Replicates (L/min)			µ (L/min)	$\sigma^{2}$
			${\left(q_{leak}\right)}_{1}$	${\left(q_{leak}\right)}_{2}$	${\left(q_{leak}\right)}_{3}$		
(1)	−1	−1	0.17	0.11	0.19	0.157	0.001
ab	+1	+1	12.78	4.62	7.73	8.38	11.30
a	+1	−1	3.61	3.84	3.49	3.65	0.021
b	−1	+1	1.66	2.31	0.98	1.65	0.29

**Table 4:** Printing parameters.

Parameter	EAC	Inextensible Portion	Extensible Portion	L-Junction
Material	TPU 95 A	TPU 95 A	TPU 80 A LF	TPU 80 A LF
Flow	106%	106%	120%	120%
Infill percentage	100%	100%	100%	100%
Infill pattern	Circular	Zigzag	Lines	Lines
Temperature	225 °C	225 °C	240 °C	240 °C

**Table 5:** Displacement from rest position in x–y space for each monolithic bending PneuNets (MBP) type.

R Type		S Type		B Type		D Type	
Tip Rest Position (mm) (x = 0; y = 100)		Tip Rest Position (mm) (x = 0; y = 101.4)		Tip Rest Position (mm) (x = 0; y = 95)		Tip Rest Position (mm) (x = 0; y = 96.9)	
Pressure input (bar)	Tip displacement (x;y) (mm)	Pressure input (bar)	Tip displacement (x;y) (mm)	Pressure input (bar)	Tip displacement (x;y) (mm)	Pressure input (bar)	Tip Displacement (x;y) (mm)
0	(0;0)	0	(0;0)	0	(0;0)	0	(0;0)
1	(15.6;3.3)	1	(15.6;3.2)	1	(7.4;1.2)	1	(8.2;1)
2	(33.2;8)	2	(27.2;8)	2	(15.6;3.1)	2	(17;3.3)
3	(47.1;22.1)	3	(39.9;18)	3	(29.8;10.7)	3	(27.7;8.4)
4	(55.6;37)	4	(54.6;39.3)	4	(36.7;17.4)	4	(41.9;23.4)
